# Methicillin-Resistant *Staphylococcus aureus* (MRSA) in Poultry Species in Algeria: Long-Term Study on Prevalence and Antimicrobial Resistance

**DOI:** 10.3390/vetsci7020054

**Published:** 2020-04-27

**Authors:** Ismahane Benrabia, Taha M. Hamdi, Awad A. Shehata, Heinrich Neubauer, Gamal Wareth

**Affiliations:** 1 HASAQ Laboratory, High National Veterinary School, Issad Abbes Avenue, Oued Smar, El Harrach, Algiers 16270, Algeria; moussahamdi@hotmail.com; 2 Avian and Rabbit Diseases Department, Faculty of Veterinary Medicine, Sadat City University, Sadat 32897, Egypt; dr_awadali_1@yahoo.com; 3 Research and Development Section, PerNaturam GmbH, 56290 Gödenroth, Germany; 4 Friedrich-Loeffler-Institut, Institute of Bacterial Infections and Zoonoses (IBIZ), Naumburger Str. 96a, 07743 Jena, Germany; heinrich.neubauer@fli.de; 5 Faculty of Veterinary Medicine, Benha University, Moshtohor, Toukh 13736, Egypt

**Keywords:** antimicrobial resistance, *S. aureus*, MRSA, Algeria, poultry

## Abstract

Methicillin-resistant *Staphylococcus aureus* (MRSA) is a well-known pathogen with a serious impact on human and veterinary public health. To determine antibiotic resistance of MRSA in poultry, 4248 nasal swabs were collected from 840 poultry farms in 18 different Wilayas (provinces) of Algeria. Swabs were collected between 2011 and 2018 from breeding hens, laying hens, broilers, and turkeys. Identification was carried out by the classical culture methods, and the disc diffusion test was used to determine the antibiotic resistance patterns. *S. aureus* was isolated from 477 (56.8%) farms, and flock prevalence was 52.8%, 48.8%, 48.4%, and 75.6% in breeding hens, laying hens, broilers, and turkeys, respectively. MRSA was isolated from 252 (30%) farms and flock prevalence was 22%, 33.5%, 27.4%, and 36%, respectively. As expected, all MRSA isolates were resistant to cefoxitin, penicillin G, amoxicillin/clavulanic acid, and oxacillin. High levels of resistance were found for tetracycline (82.5%), erythromycin (70.6%), clindamycin (68.6%), and ciprofloxacin (50%). Almost all isolates were susceptible to vancomycin (100%) and mupirocin and rifampicin (99.2%), followed by chloramphenicol (82.3%), and gentamicin (76%). This moderate proportion of MRSA in poultry poses a considerable risk to public health. The results of this study highlight the need for control programs that encompass primary animal production and the food chain to mitigate contamination and spread of MRSA in the poultry industry of Algeria, and consequently to humans.

## 1. Introduction

*Staphylococcus aureus* (*S. aureus*) is a Gram-positive bacterium causing a wide variety of suppurative infections in both humans and animals. In humans, it is one of the most common causes of bacteremia, skin, and soft tissue infection [1,2]. In animals, it is one of the most prevalent causative agents of clinical and subclinical mastitis in dairy farms, causing approximately one-third of cases in cattle, resulting in significant economic losses [3]. In Algeria, research focuses on clinical disease, e.g., mastitis in sheep [4] and nasal carriage of *S. aureus* strains, such as the highly pathogenic clones ST80 or ST152 PVL+ [5,6]. In poultry, *S. aureus* is a ubiquitous pathogen found in feedstuff as well as on equipment and utensils present in farms. It is considered an important cause of omphalitis, gangrenous dermatitis, and localized abscesses. It can also be spread via the bloodstream resulting in synovitis, chondronecrosis with osteomyelitis, arthritis, and endocarditis [7,8,9]. 

Methicillin-resistant *S. aureus* (MRSA) isolates are resistant to the majority of the antibiotics used to treat *Staphylococcus* infections in humans. It is a significant cause of severe nosocomial infections and a major public health concern globally [10]. Multidrug-resistant (MDR) Panton-Valentine Leukocidin-Positive MRSA was isolated from humans in Algeria and is being targeted for control by the Algerian health care system [5]. The proportion of clinical MRSA isolates increased from 4.8% in 1997 to 52% in 2009 [11], with a small reduction to 47% in 2016 [12]. 

The investigation of the risk of human infection following contact with animals or their products, thereof, is still neglected in Algeria. The widespread, unrestricted use of antimicrobial compounds in food-producing animals in North African countries has led to the emergence of MDR, making control and eradication of MRSA difficult. MRSA strains have been isolated from several types of food products, including raw and cooked meat, dairy products from Egypt [13], Tunisia [14], and Morocco [15]. In Tunisia, 1.2% of raw chicken meat samples were MRSA positive, and in Egypt, 38% of retail raw chicken samples were *mec*A PCR positive [16]. In Algeria, MRSA that carried the *mec*A gene was isolated from raw milk and traditional dairy products [17]. A high prevalence has also been reported from raw food products in Western Algeria [18]. 

Antimicrobial susceptibility of *S. aureus* is commonly investigated in humans in Algeria. A few studies have been conducted on antimicrobial resistance (AMR) in *S. aureus* isolates of milk-producing livestock, but little attention has been given to MRSA in poultry. The purpose of this study was to determine the prevalence and the antimicrobial profile of MRSA strains isolated from poultry species (breeding hens, laying hens, broiler, and turkey) in 18 different Wilayas (provinces) of Algeria in the period between 2011 and 2018.

## 2. Materials and Methods 

### 2.1. Sampling and Farm Information

**Figure 1:**
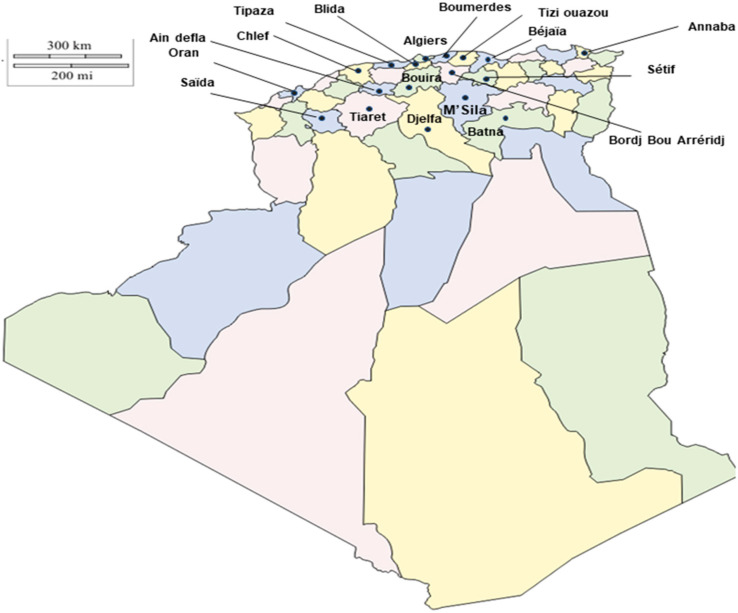
Map of Algeria displaying the 18 provinces sampled for *S. aureus* and Methicillin-resistant *Staphylococcus aureus* (MRSA) prevalence in poultry farms.

A long-term survey of *S. aureus* and MRSA was conducted in different districts in Algeria. Between 2011 and 2018, a total of 840 poultry farms were visited and sampled. Different farms were sampled each time. Before sampling, approval was obtained from the Ethical Committee at the Office of the Dean at High National Veterinary School, as well as obtaining permission from the farm owners. The farms were located in 18 different Wilayas (provinces) of Algeria (Figure 1). Sampling was throughout the calendar years 2011–2018. Five to six nasal swabs were aseptically collected and sent to the laboratory. Bacterial identification and assessment of antibiotic resistance patterns were carried out at the National Institute of Criminalistics and Criminology (INCC), Algeria. In total, 4248 nasal swabs were collected from breeding hens (n = 654), laying hens (n = 838), broilers (n = 1614), and turkeys (n = 1142). 

### 2.2. Isolation and Identification of S. aureus and MRSA

All 4248 swabs were placed in 50 mL broth containing 1% tryptone, 7.5% sodium chloride, 1% mannitol, and 0.25% yeast extract, followed by incubation at 37 °C for 24–48 h. After incubation, a loopful of broth of all 4248 samples showing turbidity or not were transferred onto Chapman agar (Bio-Rad, Marnes-la-Coquette, France) and selective MRSA agar plates (BD BBL CHROM agar MRSA, Paris, France), and then incubated at 37 °C for 24–48 h. Only isolates showing growth on both Chapman and MRSA plates were considered MRSA isolates and screened for coagulase activity (Rabbit plasma, Oxoid, Dardilly, France) and identified by the API Staph ID test (BioMerieux, Craponne, France), as described elsewhere, using a single colony from MRSA plates [19,20]. Isolates with confirmed coagulase activity and identified by the API Staph ID were considered as MRSA positive strains. According to the inclusion criteria, Chapman agar negative CHROM agar MRSA positive isolates from 23 farms were excluded from the study. *S. aureus* isolates grown on Chapman agar but not on CHROM agar were confirmed by the API Staph ID test.

### 2.3. Antimicrobial Susceptibility Testing (AST)

In total, 252 MRSA isolates (one isolate/positive farm) were tested. Only one isolate was included from each positive farm due to scarce resources and funding. The antibiotic susceptibility testing (AST) was performed by the disk diffusion technique according to the guidelines of the Clinical Laboratory Standards Institute (CLSI, 2018). A maximum of four discs was placed on each plate. The following antibiotics disks (Oxoid) were used: penicillin G (PG/10 UI), amoxicillin/clavulanic acid (20/10 µg), oxacillin (1 µg), cefoxitin (30 μg), gentamicin (10 μg), tetracycline (30 μg), vancomycin (30 μg), ciprofloxacin (5 μg), streptomycin (500 μg), trimethoprim/sulphamethoxazole (1.25 μg/23.75 μg), kanamycin (30 μg), chloramphenicol (30 μg), erythromycin (15 μg), mupirocin (200 μg and 5 μg), clindamycin (2 μg), and rifampicin (5 μg). The minimum inhibitory concentration (MIC) for cefoxitin and mupirocin was determined with E-test strips (AB BioMerieux, Marcy l′Etoile, France), according to the manufacturer’s instructions. *S. aureus* strains ATCC25923 and ATCC29213 were used as quality control strains for disk diffusion and MIC determination, respectively. 

## 3. Results

Samples were collected from 18 out of 48 Wilayas (provinces) in Algeria. In total, 1276 isolates of *S. aureus* were obtained from 4248 nasal swabs. In total, 195 isolates were recovered from breeding hens, 262 isolates from laying hens, 478 isolates from broilers, and 341 isolates from turkeys. As shown in Table 1 (Supplementary Table S1 containing the metddata and antimicrobial resistance of MRSA isolates), *S. aureus* was detected in 477 farms (56.8%). The highest prevalence was found on turkey farms (73.6%), followed by breeding hens (52.8%), laying hens (48.8%), and broilers (48.4%). MRSA was detected in 252 farms (30%), and the highest prevalence was that of turkey farms (36%). Most often, one or two MRSA were recovered on each farm. In total, 252 isolates tested (one isolate/positive farm) were resistant to cefoxitin and thus were confirmed as MRSA.

The temporal distribution of MRSA positive farms from 2011–2018 revealed that the highest rate of MRSA positive farms was reported in 2015 (n = 45 farms), 2014 (n = 37 farms), and 2016 (n = 35 farms), while in 2018 only 16 farms were MRSA positive (Table 2). There was neither an increase in prevalence at the flock level nor an increase in resistance against the tested antimicrobial compounds or a change in the patterns (Table 3). The prevalence did vary by the poultry species in the results obtained. The percentages of *Staphylococcus aureus* and MRSA positive flocks in the different poultry species varied during the period our study took place. The highest number of MRSA positive farms was observed in Algiers with 45 farms, followed by Boumerdes with 42 farms, Bordj Bou Arreridj with 33 farms, Bouira with 25 farms, and Djelfa with 19 farms. On the other hand, the provinces of Oran, Chilef, and M’sila were represented by only one farm each.

As shown in Table 4 and as expected, 100% of the MRSA isolates (n = 252) were resistant to penicillin G, amoxicillin/clavulanic acid, oxacillin, and cefoxitin. The isolates were also highly resistant to tetracycline (82.5%), erythromycin (70.6%), clindamycin (68.6%), and ciprofloxacin (50%). All MRSA isolates recovered from breeding and laying hens, as well as from broilers and turkeys, were susceptible to vancomycin, mupirocin, and rifampicin, with the exception of two isolates isolated from turkeys that showed resistance to the latter. Around 82.3% of isolates were susceptible to chloramphenicol, 76.1% to gentamicin, 75.8% to streptomycin, 72.6% to trimethoprim/sulfamethoxazole, and 58.73% to kanamycin. About half of the MRSA isolates (n = 125) were susceptible to ciprofloxacin. All isolates were multidrug-resistant. All MRSA isolates (n = 252) exhibited the antibiotic resistance (AR) pattern PenAmcOxaFox. Most of isolates 82.5% (n = 208) exhibited the pattern PenAmcOxaFox-Tcy, and 113 isolates (49%) exhibited the pattern PenAmcOxaFox-EryCln. Twenty-three isolates (9%) exhibited the pattern PenAmcOxaFox-TcyCepStrEry, and thirteen isolates (5%) exhibited the pattern PenAmcOxaFox-CepStrSxtKanCamEry, among them, eight isolates were resistant to gentamicin. Only two isolates exhibited the pattern PenAmcOxaFox-MupRif. See Table 4 for the key to abbreviations.

The metadata and antimicrobial patterns of all MRSA isolates in the current study to a panel of 16 antibiotics are shown in Supplementary Table S1.

## 4. Discussion

*Staphylococcus aureus* is a contagious, opportunistic pathogen that causes clinical or subclinical infections in humans and animals, and it is a significant cause of foodborne illness worldwide. In Algeria, a high prevalence of *S. aureus* and multi-drug resistant MRSA is found regularly in patients admitted to healthcare facilities [11,21,22,23,24,25,26,27,28,29]. Enterotoxigenic *S. aureus* and MRSA have also been isolated from samples of various raw and processed foods, including products of poultry with a contamination rate between 6.1% and 38% [17,18,30]. In food products from Western Algeria, 21.5% of *S. aureus* strains isolated, including those from poultry, were found to be MRSA [18]. Thus, it is thought that poultry primary production is a potential reservoir for MRSA foodborne infections. Only one previous prevalence study on MRSA in the poultry industry in Algeria is available [31]. They found that 42% and 12% of the laying hens and the broilers carried *S. aureus,* respectively, and more than 50% of isolates were MRSA. Although the authors investigated 1875 and 6500 samples from laying hens and broilers from six provinces over five years, they failed to record flock prevalence for MRSA and prevalences within the flock. Therefore, in the current study, we focused on determining flock prevalences to gain preliminary data for future risk assessment studies for the food production sector and associated occupational risks. In most MRSA positive flocks, only one or at most two MRSA isolates were detected. Testing only one isolate from each farm due to limited resources is a significant limitation of the study as results may not be representative of the full range of MRSA present on Algerian poultry farms.

The flock prevalence of *S. aureus* of 57% (477 poultry farms affected in 18 provinces) and of MRSA of 30% (252 farms) was unexpectedly high in our study when compared to previous studies [6,31] in Algeria. Additionally, we included turkey farms and farms of breeding hens to gain a more comprehensive overview of the primary production of poultry in Northern Algeria. Interestingly, the highest prevalence was found in turkeys. The reasons for this finding have to be investigated in future studies, but a common source of infected chickens or widespread abuse of antibiotics in this production type may be possible explanations. The rates of MRSA flock prevalence in our study were lower than those previously reported in laying hens (57%) and broiler chickens (53%) [31]. Both studies cannot be compared due to methodological differences, e.g., pre-enrichment or incubation times. It seems that the rate of MRSA positive flocks per year fluctuates and is not constant for all production areas. This requires further study.

MRSA colonization can pose an occupational risk. MRSA colonization rates of 27.78% were reported in poultry farmers in Malaysia [32]. Even in countries with a highly developed poultry industry and restriction on the use of antibiotics, such as the Netherlands, the prevalence of livestock-associated MRSA can be as high as 5.6% in specimens obtained from poultry slaughterhouse workers [33]. Unfortunately, the risk of human infection following animal contact is neglected in Algeria. Our data show that MRSA is widely distributed in the poultry industry of Algeria and that the risk of livestock acquired infection exists for consumers and people working in the food industry. Increases in human MRSA infection rates have been reported from several other North African countries [34]. In Tunisia, the prevalence of MRSA increased between 2002 and 2007 from 16% to 41%, and the prevalence was 31% in 2007 in Libya. In Egypt, multicenter studies reported a prevalence of 52% between 2003 and 2005, and a similar rate of 55% was observed in Ethiopia. However, the data have to be interpreted with care as they are biased by a low number of samples, study areas, and limited technical resources. The impact of MRSA may also vary significantly depending on the monitoring system and hygiene programs in place. MRSA control measures at the hospital level have promoted a reduction in the incidence of MRSA in the United States and several European countries.

The antibiotics most often used during the study period on the poultry farms investigated were quinolones and fluoroquinolone (oxolinic acid, flumequine, and enrofloxacin), tetracyclines (doxycycline), β-lactams (amoxicillin, ampicillin), sulfonamide and trimethoprim, as well as tylosin in turkeys (data not shown). Vancomycin is the drug of choice for treatment of severe MRSA infections [35]. Vancomycin resistant MRSA was reported in patients and food samples from Algeria [21,30], but no resistance to vancomycin was found in this study. According to the World Health Organization (WHO), rifampicin and mupirocin are critical antibiotics. Mupirocin is widely used to prevent nasal colonization of *S. aureus* in humans. Its prolonged and frequently unjustified use in humans has led to the emergence of mupirocin-resistant (mupR) *S. aureus* in Africa [36]. The use of mupirocin is limited in veterinary medicine in some countries, but cases of high-level resistance to mupirocin have been reported in MRSA isolates from dogs in Poland [37]. In the present study, two mupirocin MRSA strains were isolated from turkeys. No data on MRSA resistance to mupirocin was available for Algeria before this study. These findings are similar to those from Germany, where resistance to mupirocin was also found in 2.3% isolates from turkeys [38]. Low resistance rates of 2% for rifampin are not unexpected but were restricted again to isolates from turkeys. It may be suspected that rifampicin and mupirocin resistant strains were a spill over from humans to animals, and they may generate a special risk when multiplying in livestock. Thus, molecular typing of MRSA isolates obtained from poultry is necessary to trace back the source of infection and to compare with the isolates recovered from humans in Algeria.

Besides the resistance against beta-lactams, notable resistance against gentamicin, erythromycin, clindamycin, and tetracyclines were found. The resistance against gentamicin was again higher in MRSA isolates from turkeys. Similar results were reported from Germany with resistance rates of up to 26.1% in turkeys vs. 2.9% in those from broilers [38]. Other authors from Algeria reported a resistance rate to gentamicin of 39% in MRSA isolated from food samples, but only a few strains were investigated so that comparison with to data from this study is not appropriate [18]. In MRSA, resistance rates of over 70% for erythromycin and clindamycin are not unusual worldwide; the mechanisms of resistance are related. It is well known that resistance up to 30% in MRSA against tetracycline can be found in patients following admission to hospitals in Algeria [21]. But 85% resistance to the broad-spectrum tetracycline antibiotics in all poultry production areas is alarmingly high. The widespread use of tetracyclines in animal production has to be considered as the main reason for this finding. The development of resistance is to be expected in other bacterial genera as well. Resistance against ciprofloxacin in hospital-acquired MRSA strains is not unexpected. However, the reasons for the high resistance rate of 50% in poultry associated MRSA should be investigated in the future, in particular the acquisition of genomic data for such strains to identify their origin.

Many factors have to be considered in dealing with the antimicrobial resistance problems in poultry farming in Algeria [39], but control of the use of antibiotics during primary production needs to be urgently implemented. Successful antibiotic therapy in poultry farming is dependent on the use of antibiograms, but also pharmacodynamics, drug interactions, costs, and waiting time have to be taken into consideration. The omission of resistance testing remains one of the major causes of therapeutic failure on the farms visited. Drugs are mainly administered via drinking water. Thus, the solubility of the preparation and the dose consumed are essential for the efficacy of the treatment. In practice, the choice of drugs depends on the availability of the products in the Algerian market. According to the Ministry of Commerce, the value of imports of medicines for veterinary use reached 22.71 million USD, i.e., 471.4 tons, with an increase of 2.33 million USD (+11.44%) over the first eight months of 2016. This is a drop of almost 4% in the tonnage of antibiotics compared to the same period of 2015 [40]. Increasing prices have forced poultry breeders to use cheaper products. The country’s import restriction in 2018 as a result of high prices, unavailability of foreign currency, and economic decline has greatly affected the availability and use of antibiotics in poultry farms and may explain the lower rate of MRSA positive farms reported in 2018 [41].

## 5. Conclusions

In conclusion, the flock prevalence of *S. aureus* of 57% and nasal carriage of MRSA on 30% of farms in 18 provinces were unexpectedly high. The high prevalence rate of *S. aureus* and MRSA in poultry farms is alarming and is a significant risk for public health. The most alarming finding is that there is resistance to classes of antibiotics, such as rifampicin and mupirocin, that are not used on poultry farms. The constant evolution of the antibiotic resistance of MRSA isolates confirmed in this study implies the need for the implementation of surveillance programs coordinating the collection of data from the hospital, community, and livestock environments. MRSA in animals has zoonotic potential and poses a real threat to public health. Continuous monitoring of antibiotic resistance and future genomic studies to gain insights into the clonal relationship of human and animals strains are required to combat the emergence and dissemination of MRSA in Algeria.

## Figures and Tables

**Table 1:** The number of *Staphylococcus aureus* and Methicillin-resistant *Staphylococcus aureus* (MRSA) positive farms (flock prevalence) by poultry species.

Poultry Species	Number of Tested Flocks	No. (%) of *S. aureus* Positive Flocks	No. (%) of MRSA Positive Flocks
Breeding hens	163	86 (52.8%)	36 (22%)
Laying hens	170	83 (48.8%)	57 (33.5%)
Broiler	277	134 (48.4%)	76 (27.5%)
Turkey	230	174 (75.6%)	83 (36%)
Total	840	477 (56.8%)	252 (30%)

**Table 2:** Temporal distribution of *Staphylococcus aureus* and MRSA isolates recovered from 840 poultry farms in Northern Algeria between from 2011 to 2018.

Species	Breeding Hens			Laying Hens			Broilers			Turkeys			No. of MRSA Positive Flocks *
Year	No. of Samples	No. of *S. Aureus* Isolates	No. of MRSA	No. of Samples	No. of *S. aureus* Isolates	No. of MRSA	No. of Samples	No. of *S. aureus* Isolates	No. of MRSA	No. of Samples	No. of *S. aureus* Isolates	No. of MRSA	
2011	94	25	4	112	36	6	173	31	8	120	34	8	26
2012	97	27	5	103	35	7	192	76	9	120	33	9	30
2013	56	12	2	100	34	6	275	86	13	157	36	13	34
2014	87	24	5	102	30	8	289	85	12	160	35	12	37
2015	76	22	5	108	32	8	270	84	16	180	68	16	45
2016	86	28	5	100	31	6	250	86	12	150	46	12	35
2017	102	40	9	106	32	8	165	30	6	130	43	6	29
2018	56	13	1	107	32	8	0	0	0	125	46	7	16
Total	654	195	36	838	262	57	1614	478	76	1142	341	83	252

* Only one MRSA isolate per flock was tested using antimicrobial susceptibility testing.

**Table 3:** Temporal distribution of *Staphylococcus aureus* and MRSA culture-positive poultry farms in Northern Algeria between from 2011 to 2018.

Species	Breeding Hens			Laying Hens			Broilers			Turkeys			Total No. of MRSA Positive Flocks
Year	No. of Tested Flocks	No.(%) of *S. aureus* Positive Flocks	No.(%) of MRSA Positive Flocks	No. of Tested Flocks	No.(%) of *S. aureus* Positive Flocks	No.(%) of MRSA Positive Flocks	No. of Tested Flocks	No.(%) of *S. aureus* Positive Flocks	No.(%) of MRSA positive Flocks	No. of Tested Flocks	No.(%) of *S. aureus* Positive Flocks	No.(%) of MRSA Positive Flocks	
2011	17	9 (52.9%)	4 (23.5%)	16	8 (50%)	6 (37.5%)	29	10 (34.5%)	8 (25.8%)	32	22 (68.7%)	8 (25%)	26
2012	30	11 (36.7%)	5 (16.7%)	15	10 (66.7%)	7 (46.7%)	43	18 (41.9%)	9 (11.9%)	26	19 (73%)	9 (34.6%)	30
2013	13	9 (69.2%)	2 (15.4%)	21	9 (42.8%)	6 (28.6%)	46	21 (45.6%)	13 (15%)	36	23 (63.9%)	13 (36%)	34
2014	25	12 (48%)	5 (20%)	17	11 (64.7%)	8 (47%)	39	24 (61.5%)	12 (14%)	23	18 (78.2%)	12 (52%)	37
2015	19	11 (57.9%)	5 (26.3%)	24	12 (50%)	8 (33.3%)	37	22 (59.5%)	16 (19%)	34	29 (85.3%)	16 (47%)	45
2016	22	12 (54.5%)	5 (22.8%)	23	10 (43.5%)	6 (26%)	45	27 (60%)	12 (14%)	25	19 (76%)	12 (48%)	35
2017	26	17 (65.3%)	9 (34.6%)	28	13 (46.4%)	8 (28.6%)	38	12 (31.6%)	6 (20%)	23	19 (82.6%)	6 (26%)	29
2018	11	5 (45.5%)	1 (9%)	26	10 (38.5%)	8 (30.8%)	0	0	0	31	25 (80.7%)	7 (26%)	16
Total	163	86 (52.8%)	36 (22%)	170	83 (48.8%)	57 (33.5%)	277	134 (48.4%)	76 (15.9%)	230	174 (75.7%)	83 (36%)	252

**Table 4:** Resistance profiles of 252 MRSA isolates (one isolate per farm) recovered from breeding hens, laying hens, broilers, and turkeys on poultry farms in Algeria from 2011 to 2018.

Poultry Species		Breeding Hens	Laying Hens	Broiler	Turkey
Number of collected nasal swabs (n = 4248)		654	838	1614	1142
Number and percentages of MRSA positive farms (n = 252)		36 (5.5%)	57 (6.8%)	76 (4.7%)	83 (7.2%)
Results of Antibiotic Susceptibility Testing (AST) for MRSA isolates: Number (%) of resistant isolates					
Antibiotics Tested	Doses	No. (%)	No. (%)	No. (%)	No. (%)
Penicillin	PEN 10 UI	36 (100%)	57 (100%)	76 (100%)	83 (100%)
Amoxicillin/clavulinic acid	AMC 20/10 µg	36 (100%)	57 (100%)	76 (100%)	84 (100%)
Oxacillin	OXA 1 µg	36 (100%)	57 (100%)	76 (100%)	85 (100%)
Cefoxitin	FOX 30 µg	36 (100%)	57 (100%)	76 (100%)	86 (100%)
Gentamicin	GEN 10 µg	7 (19.5%)	12 (21.1%)	17 (21.1%)	18 (21.7%)
Tetracycline	TCY 30 µg	30 (83.3%)	46 (84.2%)	64 (85%)	71 (86.2%)
Vancomycin	VAN 30 µg	0	0	0	0
Ciprofloxacin	CEP 5 µg	18 (50%)	28 (51.2%)	35 (46.06%)	44 (53.2%)
Streptomycin	STR 500 µg	9 (25%)	20 (26.2%)	19 (22.7%)	20 (27.2%)
Trimethoprim/sulfamethoxazole	SXT 1.25/23.75 µg	10 (27.8%)	15 (27.8%)	20 (26.3%)	23 (28.9%)
Kanamycin	KAN 30 µg	15 (41.7%)	23 (40.7%)	28 (36.8%)	37 (44.6%)
Chloramphenicol	CAM 30 µg	7 (19.5%)	7 (12.3%)	11 (14.5%)	15 (187%)
Erythromicin	ERY 15 µg	25 (69.5%)	41 (72.3%)	56 (73.7%)	62 (75.6%)
Mupirocin	MUP 5/200 µg	0	0	0	2 (1.7%)
Clindamycin	CLN 2 µg	26 (72.2%)	43 (73.1%)	44 (57.8%)	61 (74.2%)
Rifampicin	RIF 5 µg	0	0	0	2 (1.7%)

## Supplementary Materials

The following are available online at https://www.mdpi.com/2306-7381/7/2/54/s1, Table S1 containing the metadata and antimicrobial resistance of all MRSA isolates have been uploaded in an excel sheet.
